# Fatigue Assessment Strategy Using Bayesian Techniques

**DOI:** 10.3390/ma12193239

**Published:** 2019-10-03

**Authors:** Enrique Castillo, Miguel Muniz-Calvente, Alfonso Fernández-Canteli, Sergio Blasón

**Affiliations:** 1 Royal Academy of Engineering, Don Pedro 10, 28005 Madrid, Spain; enrique.castillo@unican.es; 2 Royal Academy of Sciences, Valverde 22, 28004 Madrid, Spain; 3 Department of Construction and Engineering Manufacturing, Polytechnic School of Gijón, University of Oviedo, Campus de Viesques, 33206 Asturias, Spain; afc@uniovi.es (A.F.-C.); blasonsergio@uniovi.es (S.B.)

**Keywords:** fatigue, bayesian model, openbugs software, density function, confidence bands

## Abstract

Different empirical models have been proposed in the literature to determine the fatigue strength as a function of lifetime, according to linear, parabolic, hyperbolic, exponential, and other shaped solutions. However, most of them imply a deterministic definition of the S-N field, despite the inherent scatter exhibited by the fatigue results issuing from experimental campaigns. In this work, the Bayesian theory is presented as a suitable way not only to convert deterministic into probabilistic models, but to enhance probabilistic fatigue models with the statistical distribution of the percentile curves of failure probability interpreted as their confidence bands. After a short introduction about the application of the Bayesian methodology, its advantageous implementation on an OpenSource software named OpenBUGS is presented. As a practical example, this methodology has been applied to the statistical analysis of the Maennig fatigue S-N field data using the Weibull regression model proposed by Castillo and Canteli, which allows the confidence bands of the S-N field to be determined as a function of the already available test results. Finally, a question of general interest is discussed as that concerned to the recommendable number of tests to carry out in an experimental S-N fatigue program for achieving “reliable or confident” results to be subsequently used in component design, which, generally, is not adequately and practically addressed by researchers.

## 1. Introduction

Repeated application of variable loads over time may lead to fatigue failure of real structures and components. Besides, this type of failure occurs often unexpectedly because the magnitude of the stresses acting on these components usually remains far below the static material strength. Therefore, accurate estimation of the fatigue strength of materials is crucial to ensure safe design and maintenance of structures and components. 

In the literature, there is an extensive list of models devoted to the study of material fatigue strength, which are focused on predicting the service life (*N*) in terms of a particular generalized parameter (GP), such as the equivalent range of stresses (Δ*σ*), strains (Δε) or combinations of both (Smith–Watson–Topper, etc.). However, most of the models implied are deterministic, despite the fact that one of the main characteristics of the fatigue problem is its associated uncertainty. The high variability is inherent to the fatigue phenomenon forces fatigue failure to be considered as a random phenomenon, so that only models, including random variables should be considered valid for predicting fatigue life reliably. For that reason, there are different standards and guidelines, such as ISO [1], ASTM [2] or ASM [3], that include specific sections to suggest some recommendations for the statistical analysis of the fatigue data. Furthermore, among the different probabilistic fatigue modes proposed in the literature, the Weibull probabilistic regression model proposed by Castillo–Canteli [4], the same as those proposed by Freudenthal, Gumbel and Bolotin [5,6,7,8], defines the S-N or ε-N fields as hyperbolic percentile curves representing the same probability of failure. 

Despite the satisfactory probabilistic definition of the S-N field and the robust parameter estimation provided by the model of Castillo-Canteli, as extensively proven after being applied under consideration of different driving forces to different materials [9,10,11,12], a question still remains open and repeatedly unanswered in academic forums: “How many experimental tests should be performed to achieve a suitable probabilistic definition of the S-N field?”. Alternatively, even more precisely, “[h]ow many experimental tests should be performed to define the percentiles curves and fatigue limit for a given precision and confidence level”? The question is perhaps motivated by the fact that confidence intervals of the percentiles shrink according to the increasing number of available results as in the case of an extensive experimental campaign. Nevertheless, according to the concept of confidence bands [13,14] it can be concluded that both questions are incorrectly posed, since they cannot be answered “a priori” because of the complex relation implied among the number of tests and parameter values, or even because the influence of the suitability or unsuitability of the test strategy is applied. Only after testing and data assessment, it means “a posteriori”, the problem can be formulated in the following terms: The fatigue results for a certain probability of failure and precision can be statistically provided at any time of the experimental campaign irrespective of the number of tests available, but we have to count on the results being consequently penalized as a function of the number of tests: The smaller the number of test the higher the penalty applied in the derivation of the value of the particular fatigue property estimated for a given probability of failure according to the precision required.

In the past, other procedures, as for instance the bootstrap method [15,16,17,18], were applied to face the reliability question of the S-N field, but their high computational cost together with their questionable applicability when applied to small samples have contributed them not to be extensively widespread and consequently applied in the practical current fatigue characterization. Furthermore, different attempts have been made to incorporate the Bayesian methodology to the evaluation of fatigue results and fatigue lifetime prediction [19] under low and high cycle fatigue conditions from deterministic fatigue models, although using a mathematical-statistical formulation less accessible for laboratory and practitioner engineers. In those contributions, the advantages of applying Bayesian procedures are emphasized as a way of enhancing fatigue design reliability by incorporating technological knowledge from theoretical studies and previous experimental experience in particular in the case of a scarce number of tests available [19,20].

As will be highlighted in this work, a satisfactory solution for the calculation of the confidence bands of the problem will be found by applying the Bayesian theory, the response of the approach becoming now simple, unambiguous and rigorous from a statistical viewpoint particularly when applied to a probabilistic model of assessment.

The practical application of the Bayes methodology is facilitated by means of the application of a powerful OpenSource and free software named OpenBUGS, which despite its currently limited dissemination in the engineering area, might completely transform the way in which Bayesian methods will be applied in the future. The implementation of the OpenBUGS software into the Bayesian theory is basically explained, and thereafter applied to the assessment of the fatigue S-N data provided by Maennig [21,22,23] according to the Weibull regression model proposed by Castillo and Canteli [4], which allows the confidence bands of the S-N field to be determined as a function of the already available test results. In the assessment of this practical case, not only the variability of the model parameters is calculated, but also the confidence bands for each percentile failure curves are determined. In this way, the fatigue strength or lifetime can be estimated for any probability of failure and given precision level. Finally, the main conclusions of the work are summarized. 

Furthermore, a question of general interest is discussed as that concerned to the recommendable number of tests to carry out in an experimental S-N fatigue program for achieving “reliable or confident” results to be subsequently used in component design, which, generally, is not adequately and practically addressed by researchers.

## 2. Fundamentals Bases of the Model 

In the following, a general description of the main features of the Weibull regression model proposed by Castillo-Canteli for analyzing the S-N field is made and the implementation of the OpenBugs software into the Bayesian methods are explained in order to facilitate the comprehension of the methodology proposed and its practical application to the case of fatigue data assessment.

### 2.1. The Probabilistic Fatigue Model

**Figure 1:**
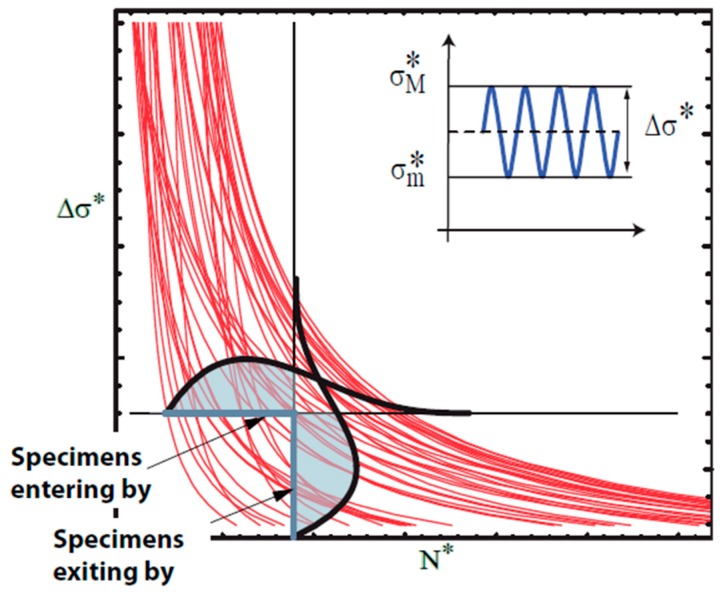
Illustration of the compatibility condition in the S-N field.

The S-N field solution for lifetime prediction proposed by Castillo and Canteli [4] is a probabilistic model, based on physical and statistical conditions to be necessarily fulfilled by any valid fatigue model, in particular on the necessary compatibility condition existing between the driving force (generally identified with the stress range, Δ*σ*) distribution for given number of cycles, and the lifetime distribution for given driving force. As shown graphically in Figure 1 for the two discretional intersecting straight lines parallel to both axes, each S-N curve pertaining to the S-N field that crosses the left part of the horizontal line must necessarily cross the lower segment of the vertical line as well, so that the areas of both shaded zones pertaining to the probability density distribution must be identical. Due to statistical considerations, see References [4,7,8], Weibull distributions are the most suitable ones for being proposed to model fitting. As a result, the following equality arises:

(1) $$E\left(N^{*},\Delta \sigma^{*}\right)=\;F\left(\Delta \sigma^{*},N^{*}\right),$$

where $E\left(N^{*},\Delta \sigma^{*}\right)$ and $F\left(\Delta \sigma^{*},N^{*}\right)$ are the cdf of the lifetime $N^{*}$ given a certain stress range $\Delta \sigma^{*}$ and the cdf of the stress range $\Delta \sigma^{*}$ given a certain fatigue lifetime $N^{*}$, respectively. Equation (1) represents a functional equation [24,25,26], which provides the only two possible solutions for the probabilistic S-N field represented by failure hyperbolic shaped percentiles curves, i.e., the curves representing the number of cycles to failure for a certain probability of failure [4]. Due to physical conditions, only the solution represented by Equation (2) is considered to be acceptable, where *N_0_* and Δ*σ_0_* are the characteristic asymptotes (limit number of cycles and fatigue endurance limit) and *λ, δ* and *β* are the location, scale and shape Weibull model parameters, respectively:

(2) $$p_{failure}=1-exp(-{\left[\frac{log\left(\frac{N}{N_{0}}\right)log\left(\frac{\Delta \sigma }{\Delta \sigma_{0}}\right)-\lambda }{\delta }\right]}^{\beta })$$

Equation (2) provides the probabilistic definition of the S-N field, i.e., the p-S-N field, which can be advantageously assessed by assuming the normalized variable $V=\;log\left(N/N_{0}\right)log\left(\Delta \sigma /\Delta \sigma_{0}\right)$. In this way, all the S-N field results are evaluated as pertaining to a single Weibull cumulative distribution function, thus, enhancing reliability in the parameter estimation. The satisfactory application of the model to practical S-N fatigue programs is confirmed for different materials and driving forces. The free and easy-to-use ProFatigue software is available to facilitate parameter estimation [27]. According to this classical concept of probabilistic approach, the five parameters of the model, i.e., *λ, δ, β, N_0_* and Δ*σ_0_*, are estimated as if they were fixed values. Nevertheless, the reliability of the parameters obtained during model fitting depends, obviously, on the total number of tests performed and the suitability of the test strategy applied in the experimental program. As a result, a sounder evaluation requires determining the densities of the estimated parameters or the confidence bands of the percentiles curves.

Until now, the calculation of the confidence intervals was achieved by applying the Bootstrap method following a procedure consisting in a previous estimation of the model parameters for the test data available and successive application of the Monte-Carlo simulation based on the model parameters just obtained following a faithful replication of the fatigue program planning adopted. The model parameters for the newly resulting simulated S-N field are estimated by applying the same probabilistic model, so that, the remaining outgoing simulated S-N fields provide the variability field of the model parameters (confidence bands) or, alternatively, the confidence bans for the particular percentile curve of interest. However, this method is usually computationally expensive and does not always report satisfactory results. As an alternative, Bayes’ methodology and its efficiency are investigated here, when applied to an advanced model, as that represented by the probabilistic S-N field solution proposed by Castillo and Canteli corresponding to Equation (2).

### 2.2. Bayesian Methods

First of all, it should be noted that the main difference between conventional and Bayesian statistical models is that the formers ones accept the model parameter estimates as fixed or deterministic values, whereas the second ones consider them as random variables. This applies to both deterministic and probabilistic models. In the application of Bayesian methods, an initial family of parametric models is assumed in which the parameters are considered to be random variables rather than constants. This means that deterministic models are transformed automatically in probabilistic ones, so they are improved. The probability distribution for each of the parameters involved, i.e., *λ, δ, β,* *N_0_* and Δ*σ_0_* is the goal to be achieved [5] when Bayesian methods are applied to the probabilistic S-N fatigue model considered in Section 2.1, and therefore, not their point estimations. In the following, the Bayesian methodology is shortly introduced for highlighting its application to fatigue model, including a brief description of the four fundamental steps to be applied, namely, (a) definition of the prior distribution, (b) estimation of the prior predictive distribution, (c) derivation of the posterior distribution and (d) calculation of the posterior predictive distribution.

#### 2.2.1. Prior Distribution

Bayesian methods are initiated assuming a prior distribution of the parameter, which may reflect the initial engineer’s knowledge about the parameters of the model and their uncertainty. This distribution can be “non-informative”, when there is no information about the parameters, or “informative”, when it contains some knowledge based on previous experience.

For example, a parametric model with a parameter vector *θ* is defined to represent a variable *X* taking *x* values, such as:

(3) X~p(x|θ).

The parameters *θ* of this function are random and are associated with a *prior distribution function*:

(4) θ~p(θ|α),

where *α* is a vector of the parameter distributions, called hyper-parameter to be distinguished from the initial model parameters (*θ*). The prior distribution may be discrete, continuous or mixed, depending on the type of parameters used.

#### 2.2.2. The Prior Predictive Distribution

Based on the description of the model and the distribution functions of the prior parameters already defined, it is possible to use the model to make predictions. Based on the description of the model and the distribution functions of the prior parameters already defined, it is possible to use the model to make predictions $\tilde{x}$:

(5) $$p\left(\tilde{x}|\theta \right)=\overset{}{\underset{\theta }{\int }}p\left(\tilde{X}|\theta \right)p\left(\theta |\alpha \right)d\theta$$

It is worth mentioning that this model only contains the information provided by the scientist or engineer knowledge on the variability of the model parameters without including any information related to evidence shown in experimental results.

#### 2.2.3. The Posterior Distribution

The knowledge about the parameters is complemented by random samples, which lead to a *posterior distribution* of the parameters, including the two sources of knowledge. Thus, once a sample of size *n* is obtained from the population to be modelled:

(6) $$X=\left(x_{1},x_{2},\ldots ,x_{n}\right).$$

It is possible to improve the predictions made by applying the maximum likelihood method by combining the prior distribution and the information provided by the sample:

(7) p(X|θ)≡L(X|θ).

At this point, the *posterior distribution* of the parameters may be derived by means of the Bayes theorem expressed by the following formula:

(8) $$p\left(X|\theta ,\;\alpha \right)=\frac{p\left(X|\theta \right)p\left(\theta |\alpha \right)}{p\left(X|\alpha \right)}.$$

#### 2.2.4. The Posterior Predictive Distribution

Finally, the *posterior predictive distribution* is obtained using the posterior distribution from the previous step, which includes both the prior provided information and the experimental evidence collected in the posterior distribution of the model parameters, thus, allowing predictions ($\tilde{x}$) to be made:

(9) $$p\left(\tilde{x}|X,\;\alpha \right)=\overset{}{\underset{\theta }{\int }}p\left(\tilde{X}|\theta \right)p\left(\theta |X,\alpha \right)d\theta .$$

Note that Bayesian models do not work with the initial family of distributions, but with the predictive distribution, i.e., a convex combination, possibly infinite, of a set of models from this family, whose weights or coefficients arise from the posterior distribution. In this way, an extended family of the initially selected family of distributions is found. This allows the sample to achieve a better model fit. Note that the resulting models are extensions of the initially assumed family of models because by considering Dirac distributions as posterior distributions, the models of the initial family are obtained.

This extension plays a significant role in the real engineering practice because it provides high flexibility in building models allowing better correlation between theoretical models and the reality to be attained as pursued in modelling fatigue problems.

#### 2.2.5. Application Example

With the aim of illustrating the different distributions, i.e., prior, prior predictive, posterior and posterior predictive distributions presented in the previous subsections, an example of the application of Bayesian Methods is presented, in which a very simplified model that follows a normal random distribution *N*(*θ*,1) is assumed. 

**Figure 2:**
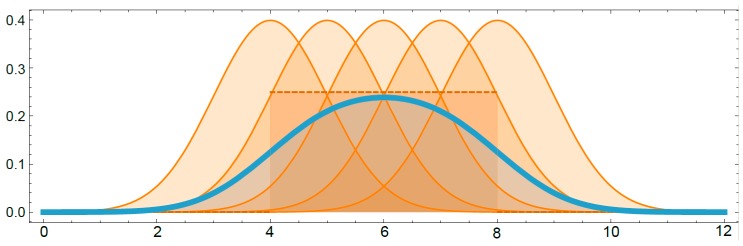
An example of a normal *N*(*θ*,1) model with a uniform *U*(4,8) prior distribution of *θ*.

In this example, the normal random model only implies one internal vector of parameters, *θ*, which is assumed to be random as well following a uniform distribution *θ~U*(4,8) (see dot line in Figure 2). As previously described, the initial random distribution of the parameters represents the *prior distribution*, and its parameters are called *hyperparameters*. Therefore, the prior distribution in this example would be the uniform distribution, and the hyperparameters will be 4 and 8.

Once the model and the prior distribution have been defined (*θ*~*U*(4,8)), it is possible to obtain the prior predictive distribution (see blue line in Figure 2):

(10) $$p\left(\tilde{x}|4,8\right)=\int_{\theta }f_{U\left(4,8\right)}\left(\theta \right)f_{N\left(\theta ,1\right)}\left(\tilde{x}\right)d\theta .$$

**Figure 3:**
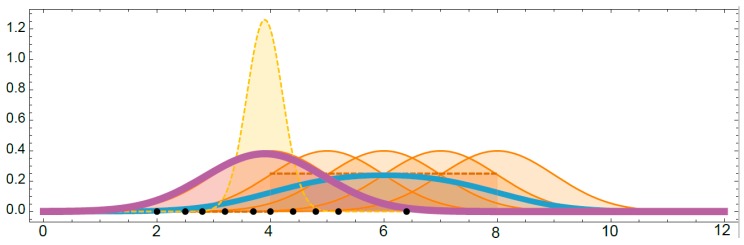
Illustration of obtaining the posterior distribution by updating the prior distribution using a sample of data.

After that, a sample of size n = 10 is obtained (see points on the horizontal axis in Figure 3) and the prior distribution improved, resulting in a posterior distribution of the parameters (see dot line in Figure 3),

(11) $$p\left(\theta |X,U\left(4,8\right)\right)\propto \prod_{i=1,10}f_{N\left(\theta ,1\right)}\left(x_{i}\right)f_{U\left(4,8\right)}\left(\theta \right).$$

Finally, the posterior predictive distribution is obtained (see violet line in Figure 3), which can be considered the final predictive model:

(12) $$p\left(\tilde{x}|X,U\left(4,8\right)\right)=\int_{\theta }f_{N\left(\theta ,1\right)}\left(\tilde{x}\right)p\left(\theta |X,U\left(4,8\right)\right)d\theta .$$

As noted, Bayesian methods consider parameters (*θ*) as random variables and subsequently obtain convex combinations of the initial model family. In fact, the distribution, actually assumed at the end of the method, is a weighted or mixed distribution, which results after combining the prior or posterior distribution with the initial distributions family.

## 3. The Proposed Model

In this section, the application of the Bayesian method to the probabilistic S-N field model of Castillo–Canteli, see Equation (2), is presented with the five parameters of the model being considered as random variables. To this end, the OpenBUGS software package has been used, which, given prior distributions and a sample of experimental results, generates large samples of the posterior model based on Markov Chain Monte Carlo (MCMC) techniques. This means that a large sample of any statistic can be accomplished and, consequently, an excellent approximation of its probability distribution is achieved. 

On the following, the implementation of the Bayesian Weibull fatigue Model in OpenBUGS is introduced in its two variants: Code text in flat format and visual programming by the Doodle module.

### 3.1. Model Implementation Through Openbugs Code

The only extreme value distribution implemented in OpenBUGS is the generalized extreme value for maxima; so that the Castillo-Canteli model, which is based on a Weibull extreme value for minima, must be implemented by this function (See Appendix A). 

Once the model is defined, the implied parameters (*N_0_, Δσ_0_, β, λ, δ*) are defined in the prior as uniform random ones, ranging between a minimum and a maximum value:

(13) $$N_{0}\;\sim \;U\left(N_{0_{min}\;},N_{0_{max}\;}\right),\;\Delta \sigma_{0}\;\sim \;U\left(\Delta \sigma_{0_{min}\;},\Delta \sigma_{0_{max}\;}\right),\;\beta \;\sim \;U\left(\beta_{min\;},\beta_{max\;}\right),\;\lambda \;\sim \;U\left(\lambda_{min\;},\lambda_{max\;}\right),\;\delta \;\sim \;U\left(\delta_{min\;},\delta_{max\;}\right).$$

Equation (13) are the prior distributions associated with the five parameters of the Castillo-Canteli model. The values of these hyperparameters have been established in a limited range of the neighbourhoods of *N_0cc_, Δσ_0cc_, β_cc_, λ_cc_* and *δ_cc_* parameters as follows:

(14) $$N_{0_{min}\;}=0.7\;\;N_{0_{cc}\;};\;N_{0_{max}\;}=1.5\;\;N_{0_{cc}\;},\;\Delta \sigma_{0_{min}\;}=0.8\;\;\Delta \sigma_{0_{cc}\;};\;\Delta \sigma_{0_{max}\;}=1.2\;\;\Delta \sigma_{0_{cc}\;},\;\beta_{min\;}=\frac{\beta_{cc\;}}{1.5};\;\beta_{max\;}=1.5\;\;\beta_{cc\;};\;\lambda_{min}=\frac{\lambda_{cc}}{3};\;\lambda_{max\;}=2\;\;\lambda_{cc},\;\delta_{min\;}=\frac{\delta_{cc\;}}{2};\;\delta_{max\;}=\;1.5\;\;\delta_{cc\;},$$

where the values of *Δσ_0cc_, β_cc_, λ_cc_* and *δ_cc_* are those obtained by implementing the original version Castillo–Canteli model using, for example, the current version of the ProFatigue software [27]. Alternatively to the implementation of the Castillo–Canteli model by the generalized extreme function, the ones/zeros Poisson model trick can be used to generate the model in an easier way (see Appendix A).

### 3.2. Model Implementation Using Graphic Doodle in OpenBUGS

**Figure 4:**
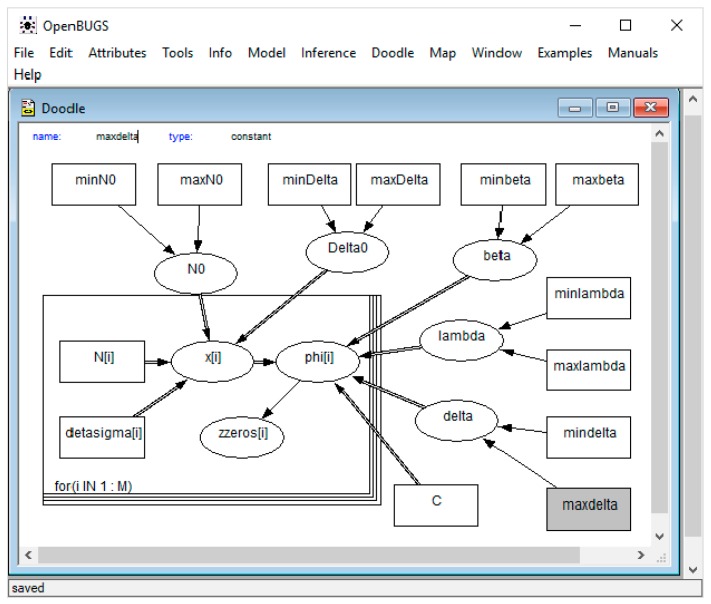
Block diagram of the Castillo and Canteli model introduced with Graphic Doodle in OpenBUGS.

OpenBUGS has a graphical programming environment called Graphic Doodle, which can be used to program the previous code easily. Figure 4 shows the acyclic graph of the Bayesian network of the model. As can be seen, the prior distributions of the five parameters have been defined outside the model loop as in the previous case.

### 3.3. Execution of the Code in OpenBUGS and Analysis of the Results Provided by the Program

**Figure 5:**
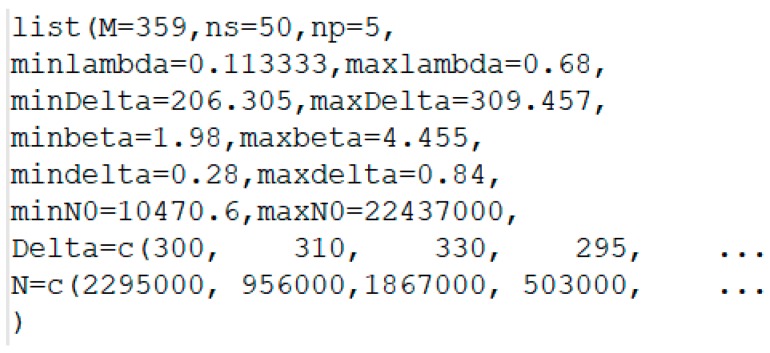
Example of definition of the initial values of the variables and the experimental fatigue data for a hypothetic fatigue case assessment.

Once the model has been implemented into the program, it is necessary to define the initial values of the variables (Equation (14)) and the experimental data of the fatigue life that to be fitted by the model, giving rise to the posterior distributions (Figure 5).

Finally, the number of simulations is defined for the initial process (burn-in process) and for the final sample, and the model is executed. In this case, taking into account the computational time of OpenBugs and the scatter associated with the fatigue problems, it is proposed to use 1,000 and 20,000 simulations for the initial and final samples, respectively.

Once the code is executed, the program provides both a convergence analysis and the model parameters of the posterior distributions, which are introduced into Equation (12) to derive the posterior predictive distribution. In this way, the percentiles of the percentiles the fatigue failure probability are obtained; in other words, the confidence intervals of each percentile as obtained by the original version of the Castillo–Canteli model.

## 4. Practical Example

In this section, the Maennig fatigue data [21,22,23], (see on Table 1), is used to illustrate the method proposed in the previous sections and its implementation in OpenBUGS. This extensive fatigue program comprising 360 results, i.e., an exceptionally high amount of data, that is not impaired, or questions the potential application of Bayesian methodology to assess fatigue programs with a limited amount of data. Its selection only obeys to a unique opportunity of observing and discussing the evolution of the confidence limits as a function of the number of results available.

**Figure 6:**
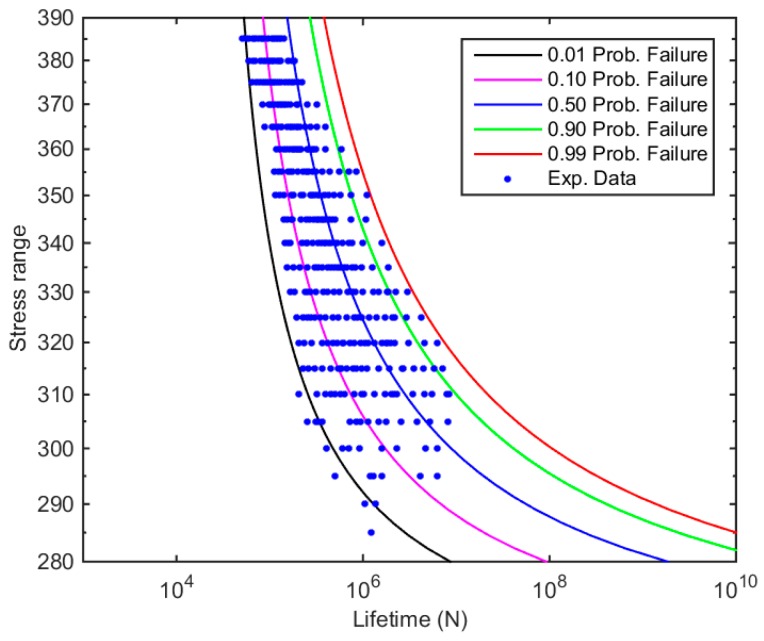
p-S-N field obtained by the standard approach of the Castillo–Canteli Model.

Firstly, the data has been fitted following the standard procedure of the Castillo–Canteli model using the ProFatigue software, in order to obtain an initial approximation of the model parameters values (see Equation (15)) and an initial estimation of the p-S-N field (see Figure 6).

(15) $$N_{0_{cc}\;}=14958\;cycles,\;\Delta \sigma_{0_{cc}\;}=257.881\;MPa,\;\beta_{cc\;}=2.97,\;\lambda_{cc}=0.34,\;\delta_{cc\;}=0.56.$$

Those values have been used to define the uniform prior distributions (Equation (13)) according to Equation (14).

**Figure 7:**
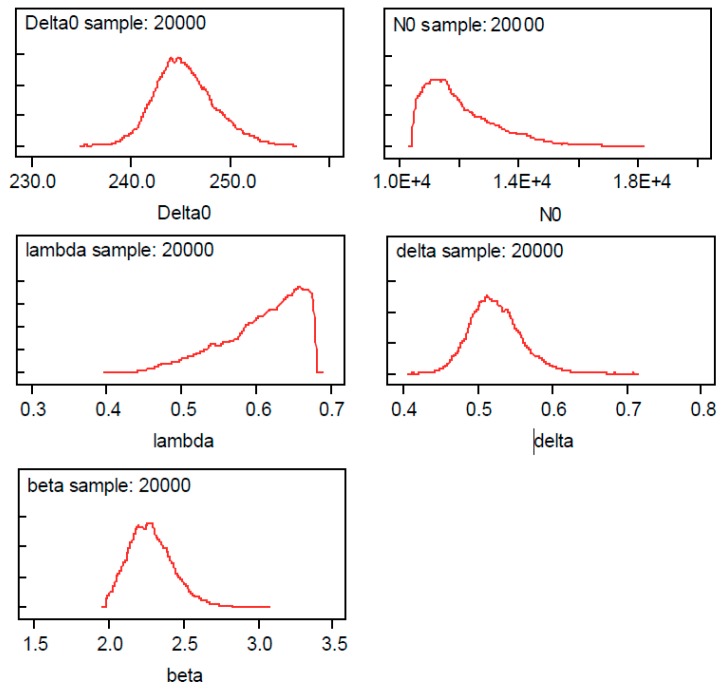
Posterior distributions of the model parameters fitted to the Maennig data.

Thereafter, data has been entered into OpenBUGS, and the simulations have been carried out to calculate the posterior distributions of the model parameters (see Figure 7).

**Figure 8:**
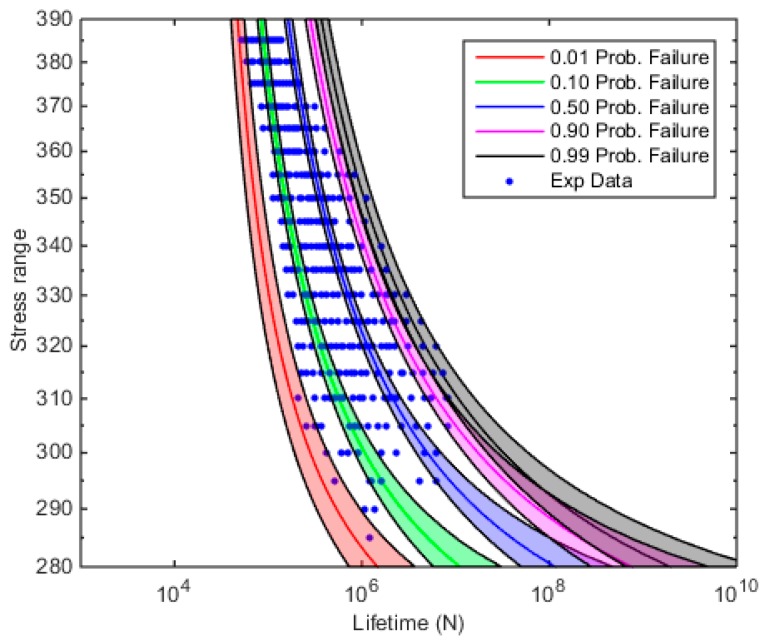
The 0.01, 0.10, 0.50, 0.90, and 0.99 quantile S-N curves and the 0.01–0.99 confidence intervals for the Maennig data.

Finally, the posterior distributions of the parameters have been used to obtain the posterior predictive distribution of the model as the 0.01, 0.10, 0.50, 0.90 and 0.99 quantiles of the S-N curves (see lines in Figure 8), and the corresponding 0.01–0.99 confidence intervals (see shaded regions in Figure 8).

### Influence of the Number of Fatigue Tests Performed

As mentioned above, the number of tests has significant influence on the results of the fitting process of a p-S-N curve. For that reason, the experimental program developed by Maennig, as an almost ideal case of fatigue data provided in the literature, may not be representative to check the suitability of the S-N assessment using the new probabilistic model. Nevertheless, the utility of the Bayes model and that of the fatigue model proposed can be checked, assuming that the virtual planning of the test program consists of limited samples, which will be randomly select from the original Maennig’s test program. The number test results considered for the assessment will be gradually increased, in order to observe their influence on the final S-N field. After their fitting, the evolution in the assessment of the fatigue program concerning the failure percentiles and fatigue limit distribution is investigated. 

**Figure 9:**
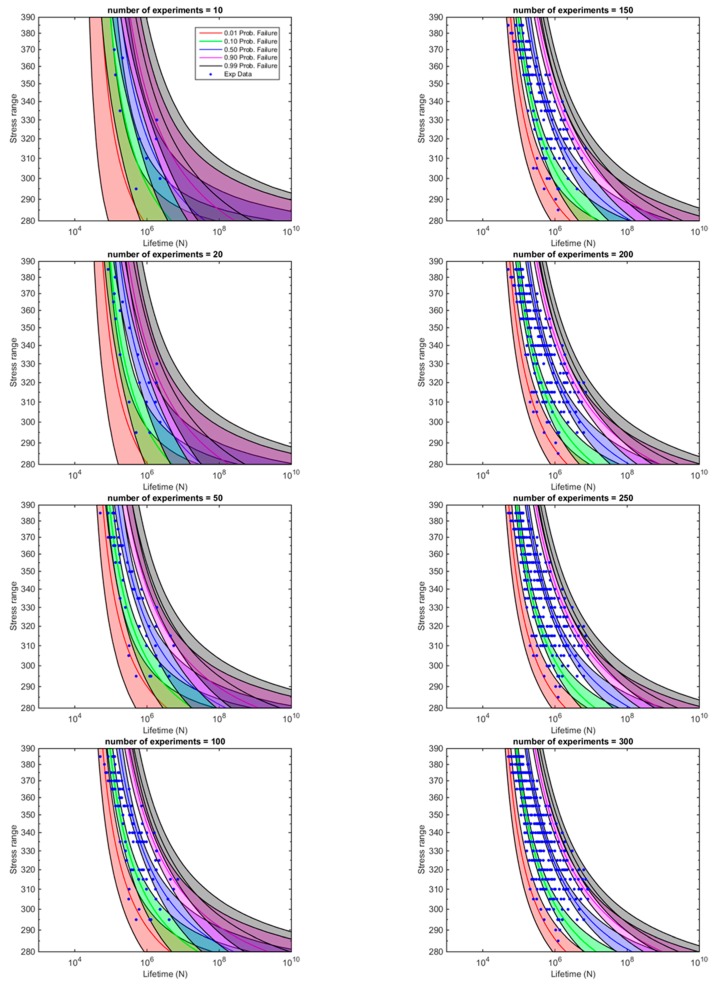
Evolution of the whole p-S-N field (S-N percentile curves and confidence intervals) with the number of tests implied in the fitting process.

To this aim, a random permutation of the 360 results contained in the vector is generated, providing a simulation of the experimental test sequence as virtually performed by Maennig. Thereafter, the Bayesian Weibull Fatigue Model is applied to fit initially only the first ten experimental results furnished by the random permutation vector (see Figure 9). The procedure is continued to define the evolution of the 0.01, 0.10, 0.50, 0.90 and 0.99 percentiles and the corresponding confidence intervals when the initial sample with 10 results is enlarged stepwise up to 20, 50, 100, 150, 200, 250 and 300 tests, respectively, as represented in Figure 9 and Video S1. As expected, the confidence intervals shrink with the number of tests, but in a non-linear way (see Figure 9).

**Figure 10:**
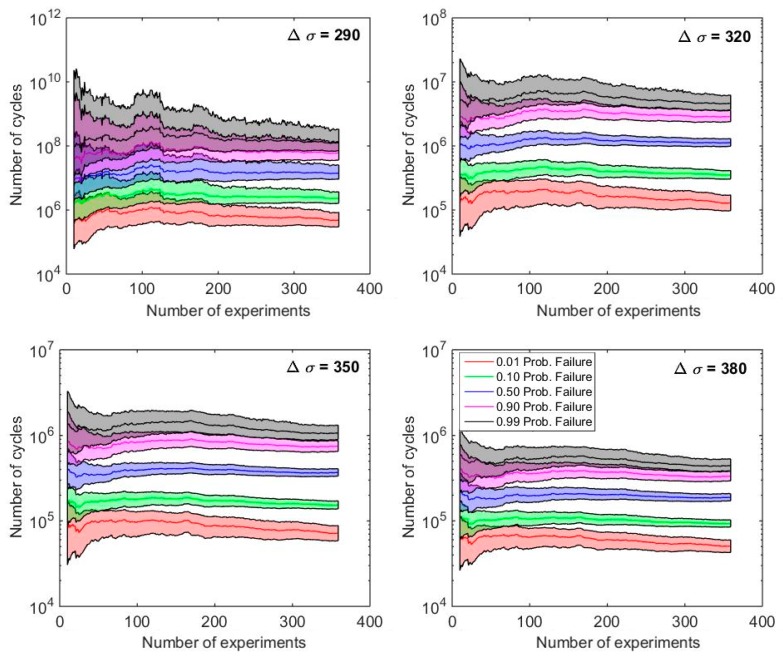
Detailed evolution of the quantiles and confidence intervals with the number of tests involved in the fitting process for different stress levels.

Despite the useful information provided by the graphs in Figure 9 showing the evolution of the confidence bands, and thus, the dependency of the whole p-S-N field with the number of cycles, a better way to observe how the confidence bands are evolving for an increasing number of fatigue data available is to y cut the p-S-N field horizontally at fixed Δ*σ* and record the number of cycles obtained for the different cases. Figure 10 represents the evolving results of the confidence bands for the 0.01, 0.1, 0.5, 0.90 and 0.99 percentiles, respectively, when such cuts are applied at Δ*σ* = 290,320,350 and 380 MPa. As expected, all confidence bands irrespective of the percentile and stress ratio considered shows a reduction for the increasing number of tests involved in the fitting process. The calculation of those graphs allows researchers to decide when the experimental running fatigue program should be finished according to a criterion of maximization of the reliability-cost rate, i.e., when a new test does not provide a profitable increment of reliability.

**Figure 11:**
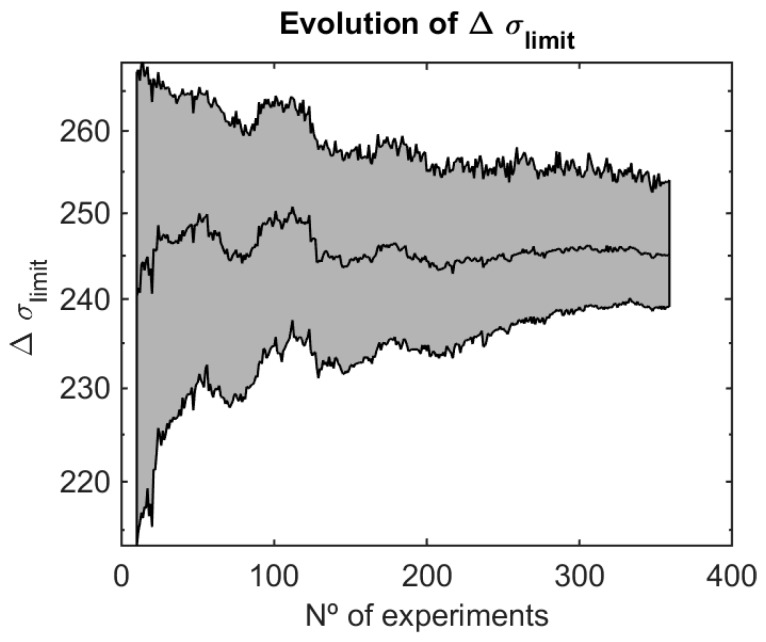
Evolution of the estimation of the fatigue limit and the 0.01–0.99 confidence intervals.

In Figure 11 the evolution of the distribution function of the fatigue limit, in particular for 0.01, 0.5, and 0.99 probability of failure is shown as a function of the total number of specimens tested by applying the Bayesian analysis. It is reminded that the fatigue limit, Δ*σ*_0_, is one of the parameters intervening in the Castillo-Canteli model, see Equation (2), representing the horizontal asymptote, i.e., the driving force below which no fatigue damage occurs. Its statistical distribution, as provided by the S-N field, has great significance in the characterization of the fatigue behavior of materials, not only as a damage lower bound, but also because its role in the interpretation of non-propagating cracks, correspondence with the threshold ΔKth in the crack growth rate curve, and determination of the intrinsic crack, but also in the definition of the Kitagawa-Takahashi diagram.

## 5. Conclusions

The main conclusions drawn from this work are the following:

(a) Concerning the Bayesian model:
  1. Despite the limited utility of standard Bayesian methods, which utility is generally restricted to specific and simple cases, the Bayesian methods based on Markov Chain Monte Carlo (MCMC) techniques, implemented in the OpenBUGS, can be advantageously used in the analysis of more complex and advanced models, particularly probabilistic ones, thus, opening the applications to a broad spectrum of fracture and fatigue problems to be explored in the future, as the one investigated here.
  2. The OpenBUGS software allows for the systematic sampling of the model parameters to be integrated into posterior predictive models, once the prior information has been enriched with the experimental data.
  3. The new Bayesian approach allows very large posterior samples of the model parameters to be obtained and use them to derive the approximate distribution of any variable instead of working with closed complex formulas.
  4. Bayesian methods, instead of providing point estimates of the variables, provide very large samples of them that can be interpreted as their density functions, which is much more than confidence intervals.
  5. The application of Bayesian techniques to the probabilistic regression Weibull model proposed by Castillo and Canteli for the analysis of S-N data enables the initial simple probabilistic definition of the five parameters model to be enhanced by providing the probability distribution for any percentile failure curve of the original model (to be interpreted as confidence intervals). Furthermore, the approach enables the subsequent evolution of the confidence intervals to be defined as a function of the number of tests carried out.
(b) Concerning the S-N model:
  1. The Bayes methodology can be applied at any time during the testing process providing an invaluable contribution to enhance the confidence intervals of the S-N assessment, in particular when using such a probabilistic model as that of Castillo-Canteli.
  2. The evolution of the confidence bands for the fatigue limit referred to the assessment of the fatigue S-N field of the Maennig example confirms the robustness of the S-N model proposed by Castillo-Canteli. In fact, the confidence bands obtained by applying the Bayes methodology proves to show a near asymptotic trend with quick diminishing for increasing number of evaluated test results evidencing a moderate variation in the ranges of the fatigue limit even for scarce number of test data.
  3. The confidence intervals in the particular case of the fatigue limit are transcendental because the direct interpretation of the latter, whereas those for the remaining S-N parameter are of less interest when separately interpreted. Instead, the reciprocal interdependence, i.e., the correlation, existing among all the model parameters is better reflected through the representation of the confidence bands (distribution of the particular probability of failure) of the percentiles S-N curves, preferably only for low or very low probability of failure in practical design.

## Figures and Tables

**Table 1:** Maennig fatigue data.

Δσ(MPa)	Lifetime (Thousands of Cycles) (*N*)
385	51,57,60,67,68,69,75,76,82,83,87,95,106,109,111,119,122,128,132,140
380	59,66,69,80,87,90,97,98,99,100,107,109,117,118,125,128,132,158,177,186
375	65,71,78,84,89,93,98,103,105,109,113,118,124,131,147,156,171,182,199,220
370	83,98,100,104,110,111,122,125,132,136,141,143,146,155,165,194,200,201,251,318
365	89,105,108,118,119,121,130,133,152,164,170,181,182,192,199,211,238,273,324,398
360	117,127,141,151,162,173,181,186,192,198,203,209,218,255,262,288,295,309,394,585
355	112,125,133,156,166,168,173,202,227,247,253,261,285,286,309,365,442,559,702,852
350	115,129,143,169,177,178,218,230,271,280,285,305,326,342,381,431,493,568,734,1101
345	140,155,169,174,218,248,265,293,321,326,348,350,364,374,397,426,461,504,738,1063
340	146,159,168,224,246,253,291,326,358,385,397,425,449,498,532,610,714,763,987,1585
335	154,180,210,254,305,332,363,415,457,482,528,559,593,611,678,767,835,957,1274,1854
330	166,184,241,251,273,312,371,418,493,562,683,760,830,981,1306,1463,1842,1867,2220,2978
325	196,227,250,271,308,347,393,475,548,669,799,879,975,1154,1388,1705,2073,2211,2925,4257
320	206,231,283,370,413,474,523,597,605,619,727,815,935,1056,1144,1336,1580,1786,1826,1943,2214, 3107,4510,6297
315	226,257,307,370,457,549,570,590,672,781,850,974,1093,1460,1477,1936,2662,2731,3487,4396,5803, 7215
310	206,317,393,446,502,570,627,809,956,1022,1327,1745,2001,2139,2314,3425,4576,5453,7868,8297
305	253,311,329,370,726,845,935,954,1139,1456,1792,2578,3776,5161,8131
300	411,606,700,707,919,1587,1595,2295,4628,6280
295	503,1191,1282,1609,4070,6337
290	1055,1369
285	1220

## Supplementary Materials

The following are available online at https://www.mdpi.com/1996-1944/12/19/3239/s1, Video S1: Evolution of the whole p-S-N field, Video S2: Evolution of the quantiles and confidence intervals with the number of tests.

## Appendix A. OpenBugs Codes

This Appendix shows the code implemented in OpenBugs to introduce the Castillo-Canteli model by using the generalized extreme function. To do that, and with the aim of facilitating the reading of the code, four auxiliary variables have been defined (lines 3 to 6), which are used to define in a simpler way the model (line 8).

Once the model is defined, the implied variables (*N_0_, Δσ_0_, β, λ, δ*) are defined as uniform random ones, ranging between a minimum and a maximum value (lines 10 to 14).

**Mode 1**

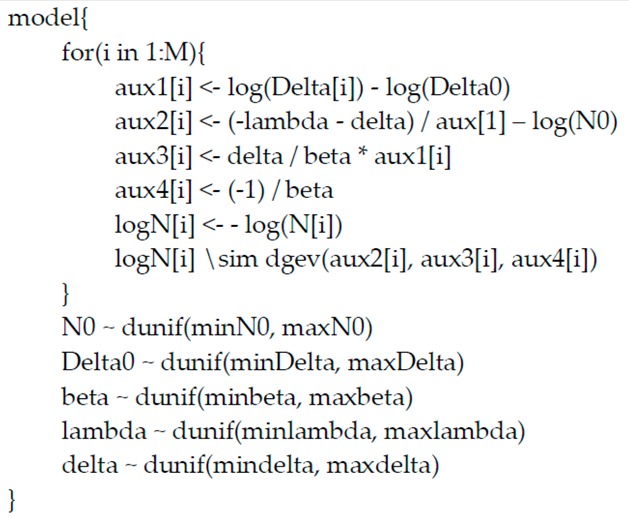

Furtheremore, the ones/zeros Poisson model trick has been used to introduce the Castillo and Canteli model, as can be seen in the following code:

**Mode 2**
